# Effectiveness of seasonal influenza vaccine for adults and children in preventing laboratory-confirmed influenza in primary care in the United Kingdom: 2015/16 end-of-season results

**DOI:** 10.2807/1560-7917.ES.2016.21.38.30348

**Published:** 2016-09-22

**Authors:** Richard Pebody, Fiona Warburton, Joanna Ellis, Nick Andrews, Alison Potts, Simon Cottrell, Jillian Johnston, Arlene Reynolds, Rory Gunson, Catherine Thompson, Monica Galiano, Chris Robertson, Rachel Byford, Naomh Gallagher, Mary Sinnathamby, Ivelina Yonova, Sameera Pathirannehelage, Matthew Donati, Catherine Moore, Simon de Lusignan, Jim McMenamin, Maria Zambon

**Affiliations:** 1 Public Health England, London, United Kingdom; 2 Health Protection Scotland, Glasgow, United Kingdom; 3 Public Health Wales, Cardiff, United Kingdom; 4 Public Health Agency Northern Ireland, Belfast, United Kingdom; 5 West of Scotland Specialist Virology Centre, Glasgow, United Kingdom; 6 University of Strathclyde, Glasgow, United Kingdom; 7 University of Surrey, Guildford, United Kingdom; 8 Royal College of General Practitioners, London, United Kingdom

**Keywords:** influenza, vaccines, immunisation

## Abstract

The United Kingdom (UK) is in the third season of introducing universal paediatric influenza vaccination with a quadrivalent live attenuated influenza vaccine (LAIV). The 2015/16 season in the UK was initially dominated by influenza A(H1N1)pdm09 and then influenza of B/Victoria lineage, not contained in that season’s adult trivalent inactivated influenza vaccine (IIV). Overall adjusted end-of-season vaccine effectiveness (VE) was 52.4% (95% confidence interval (CI): 41.0–61.6) against influenza-confirmed primary care consultation, 54.5% (95% CI: 41.6–64.5) against influenza A(H1N1)pdm09 and 54.2% (95% CI: 33.1–68.6) against influenza B. In 2–17 year-olds, adjusted VE for LAIV was 57.6% (95% CI: 25.1 to 76.0) against any influenza, 81.4% (95% CI: 39.6–94.3) against influenza B and 41.5% (95% CI: −8.5 to 68.5) against influenza A(H1N1)pdm09. These estimates demonstrate moderate to good levels of protection, particularly against influenza B in children, but relatively less against influenza A(H1N1)pdm09. Despite lineage mismatch in the trivalent IIV, adults younger than 65 years were still protected against influenza B. These results provide reassurance for the UK to continue its influenza immunisation programme planned for 2016/17.

## Introduction

The United Kingdom (UK) has had a long-standing selective inactivated influenza vaccination programme targeted at individuals at higher risk of severe disease such as the elderly, those with an underlying clinical risk condition and pregnant women. Following recommendations from the Joint Committee of Vaccination and Immunisation (JCVI) in 2012, the decision was taken for a phased introduction of a newly licensed live attenuated influenza vaccine (LAIV), ultimately offered LAIV in each season to all healthy children aged two to 16 years [1]. 2015/16 is the third season of the introduction of this new influenza vaccination programme; all healthy children aged two to four years and in school years 1 and 2 were offered a single dose of LAIV [2]. In Northern Ireland and Scotland and in selected pilot areas in England, all other older children of primary school age were also offered LAIV in 2015/16. Children aged two to 17 years in a clinical risk group were also offered LAIV, while children with a risk factor, in whom LAIV is contraindicated, were offered quadrivalent inactivated influenza vaccine (IIV). All children in a clinical risk group aged six to 23 months were offered IIV. The United States Centers for Disease Control and Prevention (US CDC) recently reported the observation that LAIV did not provide protection in children against circulating influenza strains in North America in the 2015/16 season [3]. This raised a question about the effectiveness of LAIV in children in the UK.

In the UK, the 2015/16 season started late, peaking in week 11 of 2016, with circulation initially dominated by influenza A(H1N1)pdm09 viruses. Impact mainly fell on younger adults resulting in large numbers of hospitalisations and admissions to intensive care units (ICU) [4]. Genetically, the haemagglutinin (HA) genes of A(H1N1)pdm09 viruses all belonged in subgroup 6B, the predominant clade circulating in the 2014/15 season. The later stages of the 2015/16 season were dominated by influenza B circulation, with the majority of viruses antigenically similar to B/Brisbane/60/2008, the influenza B/Victoria lineage component included in the 2015/16 northern hemisphere quadrivalent vaccine but not in the trivalent vaccine [4]. This raised questions about the protection provided by the 2015/16 trivalent vaccine, the main influenza vaccine offered to adults, and about the potential added value of switching to quadrivalent vaccine as the main vaccine of choice.

Following the mid-2015/16 season report of influenza vaccination effectiveness (VE) [5], this article presents the end-of-season estimates of influenza VE using well established systems across the four countries of the UK [6,7]. The aims of the investigation were to measure VE against laboratory-confirmed influenza by type, sub-type and clade/lineage, and to determine the effectiveness of the vaccine in children two to 17 years of age according to type of vaccine, particularly in relation to LAIV, but also IIV. In addition, we estimated the effectiveness of both LAIV and IIV in children two to 17 years of age over the three seasons since the UK introduced the LAIV programme.

## Methods

### Study population and period

The test-negative case–control (TNCC) design was used to estimate VE. The study was undertaken in five sentinel general practice surveillance networks across the UK, details of which have been outlined previously [7]. The surveillance schemes were: Royal College of General Practitioners (RCGP), Research and Surveillance Centre (RSC), Specialist Microbiology Network (SMN) England and Wales, Northern Ireland and Scotland.

The main study took place from 1 October 2015 until 1 May 2016. The study population were patients presenting to their general practitioner (GP) during the study period with an acute influenza-like illness (ILI), who the physician consented verbally to be swabbed during the consultation. A patient with ILI was defined as an individual presenting in primary care with an acute respiratory illness with physician-diagnosed fever or complaint of feverishness. GPs were asked to swab a random sample of cases up to a total of 10 per week in any one practice. Cases were patients who tested positive for influenza A or B virus by real-time PCR. Controls were patients with the same symptoms who tested negative for influenza A and B. Further details of the laboratory methods are provided below.

During the consultation, the GP completed a standard questionnaire. It collected demographic, clinical and epidemiological information from patients including potential confounders such as sex, date of birth, underlying clinical risk factors, date of onset of ILI, date of sample collection (recommended within seven days of onset) and influenza vaccination history for the 2015/16 season including date of vaccination and route of administration (intranasal/injection). In England, residence in an area where a primary school LAIV immunisation programme took place was also recorded.

A further specific sub-analysis was undertaken among children two to 17 years of age for the period 1 October 2013 until 1 May 2016. This covered the period since the introduction of LAIV in the UK. All aspects of data collection, testing and analysis were comparable over this period and are as described above.

### Laboratory methods

Sentinel GP surveillance networks sent the respiratory samples to the national laboratories as previously outlined [7]. Laboratory confirmation was made using comparable real-time PCR methods able to detect circulating influenza A and B viruses [8,9]. Positive samples were sent to the reference laboratories for genetic characterisation. Isolation of influenza viruses was tried from all PCR-positive samples using Madin-Darby canine kidney epithelial (MDCK) cells or MDCK cells containing the cDNA of human 2,6-sialtransferase (SIAT1) cells as described previously [10,11].

Antigenic characterisation was only undertaken at the PHE reference laboratory. Post-infection ferret antisera were used in haemagglutination inhibition (HI) assays with turkey red blood cells to antigenically characterise influenza A(H1N1)pdm09 and influenza B virus isolates with a haemagglutination titre ≥ 40 [12]. Reference virus strains used for HI assays for A(H1N1)pdm09 viruses included A/California/7/2009 (vaccine strain) grown in embryonated chicken eggs and other A(H1N1)pdm09 England strains grown in embryonated chicken eggs or tissue culture cells. Reference virus strains used for HI assays for influenza B viruses included B/Phuket/3073/2013 (trivalent and quadrivalent vaccine strain) and B/Brisbane/60/2008 (quadrivalent vaccine strain) together with a panel of other egg- and tissue culture-grown influenza B viruses from both the B/Yamagata/16/88-lineage and the B/Victoria/2/87 lineage. The fold difference between the homologous HI titre for the corresponding vaccine strain and the HI titre for each clinical isolate was calculated to determine antigenic similarity of clinical isolates to the vaccine strain.

Nucleotide sequencing of the haemagglutinin (HA) gene was undertaken (primer sequences available on request) for a subset of influenza A(H1N1)pdm09 and B viruses selected to be representative of the range of the patients’ age, date of sample collection, geographical location and, if performed, antigenic characterisation of the virus isolate, and phylogenetic trees were constructed with a neighbour-joining algorithm available in the Mega 6 software (http://www.megasoftware.net) [13]. The A(H1N1)pdm09 results have been previously presented [5]. HA sequences from reference strains used in the phylogenetic analysis for influenza B in this paper were obtained from GenBank: B/Malaysia/2506/2004 (CY038287), B/Jilin/20/2003 (CY033828), B/Yamagata/16/88 (CY018765), B/Victoria/2/87 (M58428), B/HongKong/330/2001 (AF532549) and from the EpiFlu database of the Global Initiative on Sharing All Influenza Data (GISAID) (Table 1).

**Table 1:** Influenza B haemagglutinin sequences obtained from GISAID used in the phylogenetic analysis

Influenza virus isolate	Segment ID/Accession number	Country	Collection date (year-month-day)	Originating laboratory	Submitting laboratory
B/Brisbane/3/2007	EPI154537	Australia	2007-Jan-01	Queensland Health Scientific Services, Queensland, Australia	WHO Collaborating Centre for Reference and Research on Influenza, Victoria, Australia
B/Stockholm/12/2011	EPI346827	Sweden	2011-Mar-28	Swedish Institute for Infectious Disease Control, Solna, Sweden	National Institute for Medical Research, London, UK
B/England/515/2014	EPI555201	United Kingdom	2014-Oct-22	Public Health England, London, UK	National Institute for Medical Research, London, UK
B/Estonia/77391/2013	EPI467120	Estonia	2013-Apr-08	Health Protection Inspectorate, Tallin, Estonia	National Institute for Medical Research, London, UK
B/Odessa/3886/2010	EPI271913	Ukraine	2010-Mar-19	Ministry of Health of Ukraine, Kiev, Ukraine	National Institute for Medical Research, London, UK
B/Phuket/3073/2013	EPI540675	Australia	2013-Nov-21	WHO Collaborating Centre for Reference and Research on Influenza, Victoria, Australia	National Institute for Medical Research, London, UK
B/Massachusetts/02/2012	EPI438406	United States	2012-Jan-01	New York Medical College, New York, US	Centers for Disease Control and Prevention, Atlanta, US
B/Wisconsin/01/2010	EPI271545	United States	2010-Feb-20	Wisconsin State Laboratory of Hygiene, Madison, US	Centers for Disease Control and Prevention, Atlanta, US
B/Hawaii/02/2010	EPI271558	United States	2010-Mar-25	State of Hawaii Department of Health, Pearl City, US	Centers for Disease Control and Prevention, Atlanta, US
B/Brisbane/60/2008	EPI172555	Australia	2008-Aug-04		Centers for Disease Control and Prevention, Atlanta, US
B/Florida/4/2006	EPI134356	United States	2006-Nov-01		Centers for Disease Control and Prevention, Atlanta, US
B/Bangladesh/3333/2007	EPI156050	Bangladesh	2007-Aug-18		Centers for Disease Control and Prevention, Atlanta, US

GISAID: Global Initiative on Sharing All Influenza Data; UK: United Kingdom; US: United States; WHO: World Health Organization.

### Statistical methods

Patients were defined as vaccinated if they had received the 2015/16 seasonal vaccine at least 14 days before first onset of ILI. Patients were excluded if they were vaccinated less than 14 days before symptom onset. If vaccinated, but date of vaccination was unknown, the median date of vaccination of those with known dates was taken instead. Patients with date of onset not known or onset more than seven days before swabbing were also excluded. A similar approach was used to undertake a pooled analysis for the 2013/14, 2014/15 and 2015/16 seasons.

The odds ratios (OR) obtained from multivariable logistic regression models were used to calculate VE with influenza laboratory results as the outcome and influenza vaccination status as the linear predictor. Influenza A(H1N1)pdm09- and influenza B-specific VE was also calculated. Samples positive for other subtypes were excluded as the numbers were too small, except for the three-season pooled analysis, which also included influenza A(H3N2). The adjusted estimates were set based on past seasons as age (age groups: 0–4, 5–17, 18–44, 45–64, ≥ 65 years), month of sample collection, residence in area where a primary school programme was in place, sex and surveillance scheme. We also explored whether being in a risk group for whom vaccination is recommended provided any evidence of confounding. For the three-year pooled analysis, year was also included in the model. All statistical analyses were carried out in Stata version 13 (StataCorp, College Station, Texas).

The HA sequences from England obtained in this study, which were also used in the phylogenetic analysis, were deposited in GISAID under the following accession numbers: EPI679258, EPI811543, EPI811551, EPI811554, EPI811562, EPI811570, EPI811578, EPI811586, EPI811594, EPI811598, EPI811606, EPI811614, EPI811622, EPI811626, EPI811629, EPI811637, EPI811645, EPI811648, EPI811656, EPI811664, EPI811671, EPI811675, EPI811683, EPI811691, EPI811699, EPI811707, EPI811715, EPI811723, EPI811726, EPI811734, EPI811742, EPI811750, EPI811758, EPI811766, EPI811774, EPI811782, EPI811788, EPI811796, EPI811799, EPI811807, EPI811815, EPI811823, EPI811831, EPI811839, EPI811842, EPI811845, EPI811853, EPI811856, EPI811864, EPI811868, EPI811876, EPI811884, EPI811891, EPI811894, EPI811898, EPI811906, EPI811909, EPI811915, EPI811916, EPI811924, EPI811932, EPI811940, EPI811944, EPI811952, EPI811958.

## Results

**Figure 1:**
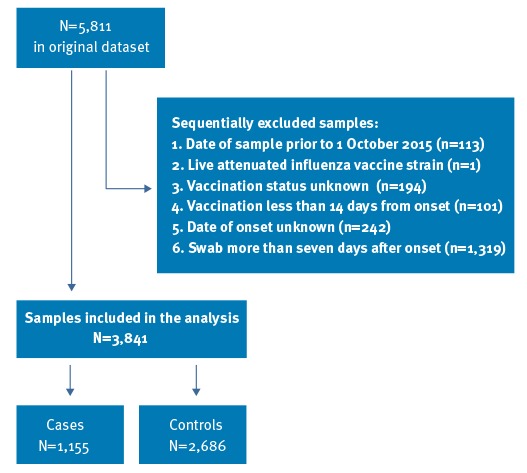
Specimen inclusion and exclusion criteria, end-of-season 2015/16 influenza vaccine effectiveness evaluation, United Kingdom, 1 October 2015–1 May 2016 (n = 5,811)

Of the 5,811 swabbed individuals potentially eligible, 3,841 individuals were confirmed eligible and included in the study (Figure 1). The details of those included in the study are provided by swab result in Table 2, including those with missing data. There were a total of 2,686 controls, 351 (9.1%) influenza B detections, 770 A(H1N1)pdm09 detections (20.0%), 24 influenza A(H3N2) detections (0.6%) and 15 influenza A(untyped) detections (0.4%). Four samples tested positive for both A(H1N1)pdm09 and influenza B and one sample was positive for both A(H1N1)pdm09 and A(H3N2). Positivity rates differed significantly by age group, sex, risk group, month, scheme, vaccination status and area of primary school programme in England (Table 2).

**Table 2:** Details for influenza A and B cases and controls, United Kingdom, October 2015–May 2016 (n = 5,811)

	Controls		Influenza B^a^		Influenza A(H1N1)^a^		Influenza A(H3N2)		Influenza A(untyped)		Total^a^	p value^b^
**Age group (years)**												
0–4	273	71.3	19	5.0	91	23.8	1	0.3	1	0.3	383	< 0.0001
5–17	392	69.3	92	16.3	78	13.8	5	0.9	1	0.2	566	
18–44	1,022	65.9	170	11.0	348	22.4	7	0.5	5	0.3	1,551	
45–64	636	70.0	47	5.2	211	23.2	7	0.8	7	0.8	908	
≥ 65	346	84.6	19	4.6	39	9.5	4	1.0	1	0.2	409	
Missing	17	70.8	4	16.7	3	12.5	0	0	0	0	24	
**Sex**												
Female	1,627	72.4	188	8.4	417	18.5	12	0.5	8	0.4	2,248	< 0.0001
Male	1,046	66.4	162	10.3	350	22.2	12	0.8	7	0.4	1,576	
Missing	13	76.5	1	5.9	3	17.6	0	0	0	0	17	
**Surveillance scheme**												
Northern Ireland	76	49.0	22	14.2	51	32.9	0	0	6	3.9	155	< 0.0001
RCGP	1,148	64.0	179	10.0	449	25.0	19	1.1	0	0	1,793	
SMN	138	67.0	12	5.8	50	24.3	1	0.5	5	2.4	206	
Scotland	1,242	81.8	101	6.6	172	11.3	3	0.2	4	0.3	1,519	
Wales	82	48.8	37	22.0	48	28.6	1	0.6	0	0	168	
**Risk group**												
No	1,794	66.5	276	10.2	607	22.5	14	0.5	9	0.3	2,697	< 0.0001
Yes	817	79.7	53	5.2	141	13.8	9	0.9	6	0.6	1,025	
Missing	75	63.0	22	18.5	22	18.5	1	0.8	0	0	119	
**Interval onset–sample (days)**												
0–1	292	67.6	41	9.5	95	22.0	2	0.5	2	0.5	432	< 0.0001
2–4	1,351	66.1	216	10.6	463	22.6	14	0.7	5	0.2	2,045	
5–7	1,043	76.5	94	6.9	212	15.5	8	0.6	8	0.6	1,364	
**Month**												
October	304	98.7	1	0.3	1	0.3	1	0.3	1	0.3	308	< 0.0001
November	396	96.1	6	1.5	8	1.9	2	0.5	0	0	412	
December	463	86.4	5	0.9	67	12.5	0	0	1	0.2	536	
January	541	68.7	26	3.3	217	27.6	3	0.4	2	0.3	787	
February	445	56.1	67	8.4	275	34.7	4	0.5	3	0.4	793	
March	366	48.0	197	25.8	190	24.9	7	0.9	5	0.7	763	
April	171	70.7	49	20.2	12	5.0	7	2.9	3	1.2	242	
**Vaccination status (all ages)**												
Unvaccinated	1,959	66.4	308	10.4	658	22.3	15	0.5	13	0.4	2,949	< 0.0001
Vaccinated (14–91 days ago)	377	89.8	6	1.4	33	7.9	3	0.7	1	0.2	420	
Vaccinated (>91 days ago)	350	74.2	37	7.8	79	16.7	6	1.3	1	0.2	472	
**Pilot area (SMN and RCGP only)**												
No	1,185	63.8	181	9.7	470	25.3	20	1.1	2	0.1	1,858	0.057
Yes	91	72.2	9	7.1	24	19.0	0	0	2	1.6	126	
Missing	11	64.7	1	5.9	4	23.5	0	0	1	5.9	17	
**Vaccine status (by route) (2–17 years)**												
Not vaccinated	402	65.5	94	15.5	112	18.2	6	1.0	1	0.2	614	0.01
Injection	16	84.2	3	15.8	0	0	0	0	0	0	19	
Intranasal	89	77.4	4	3.5	22	19.1	0	0	0	0	115	
Missing	12	70.6	1	5.9	4	23.5	0	0	0	0	17	

RCGP: Royal College of General Practitioners Research and Surveillance Centre; SMN: Specialist Microbiology Network.

Note: Differences between cases and controls for all variables in this table were statistically significant.

^a^ Four positive for influenza A(H1N1) and B; one positive for influenza A(H1N1) and A(H3N2). Duplicates are not included in row totals. 

^b^ Positive vs negative for influenza.

### Influenza A(H1N1)pdm09 and B strain characterisation from sentinel samples

Since week 40 in 2015, a total of 730 influenza viruses from this study have been characterised by the PHE Respiratory Virus Unit and the West of Scotland Virology Centre: 128 antigenically, 293 genetically and 309 through both methods. Only the PHE Respiratory Virus Unit undertook the antigenic analysis.

A total of 482 influenza A(H1N1)pdm09 viruses were characterised. All belonged in the genetic subgroup 6B, which had been the predominant genetic subgroup in the 2014/15 season. Some heterogeneity was seen in the HA of the current season’s A(H1N1)pdm09 viruses, with some newly emerging genetic subgroups: the HA genes of the majority (93%) of A(H1N1)pdm09 viruses fell into genetic cluster 6B.1, characterised by the amino acid changes S84N, S162N (with gain of a potential glycosylation site) and I216T, with a subset in this cluster having the substitution A215G. Less than 6% of viruses fell into a second emerging cluster (6B.2) and had the amino acid substitutions V152T, V173I, E491G and D501E in the HA gene, or into a third minor cluster with substitutions N129D, R450K and E491G. A few viruses from this season did not have any of these changes or had only the substitution S84N, and clustered with A(H1N1)pdm09 viruses from season 2014/15 (6B subgroup). A tree showing the phylogenetic relationships for the A(H1N1)pdm09 has already been published [5]. Of 123 A(H1N1)pdm09 viruses isolated from sentinel samples between December 2015 and April 2016 and analysed by HI assay using an extended panel of ferret post-infection sera including a ferret post-infection antiserum to A/California/7/2009 (NIBSC, UK), 100% were antigenically similar to the A/California/7/2009 northern hemisphere 2015/16 A(H1N1)pdm09 vaccine strain. Using this extended panel of ferret post-infection antisera, no antigenic low reactors to A/California/7/2009 antisera were observed.

**Figure 2:**
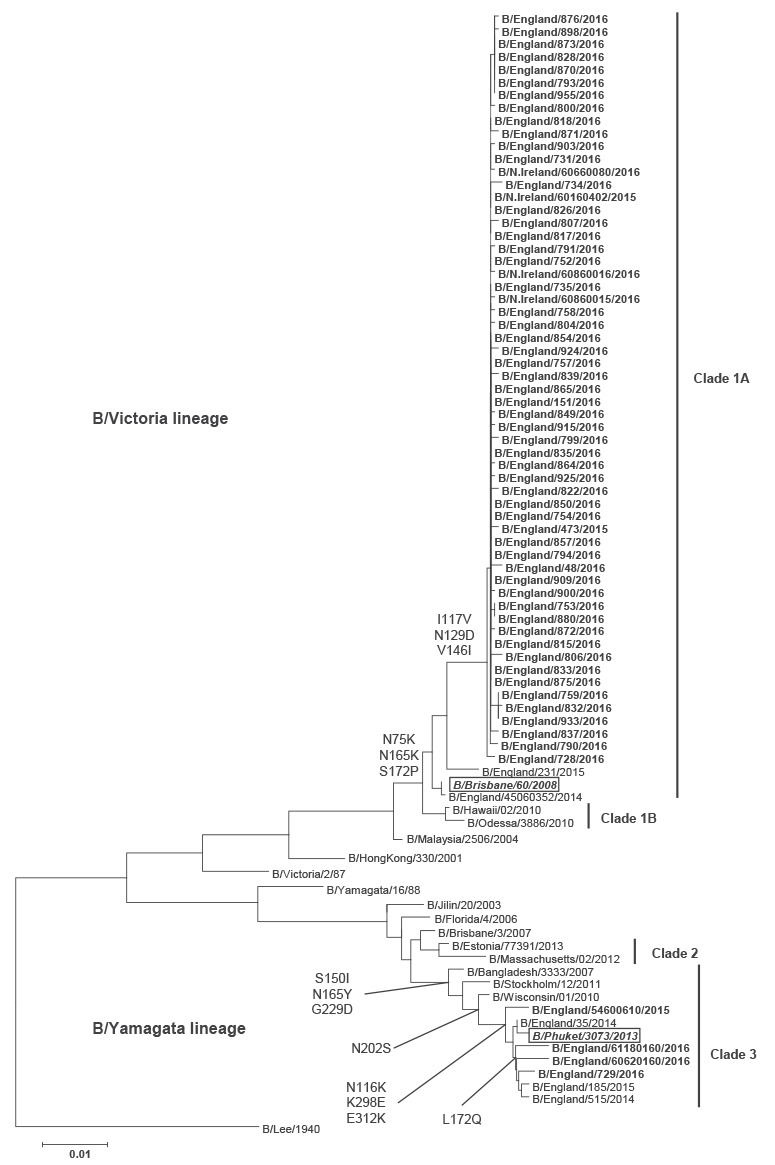
Phylogenetic tree of the haemagglutinin genes of sentinel influenza B isolates, United Kingdom, October 2015–May 2016 (n = 324)

A total of 324 influenza B viruses were characterised: more than 96% of them belonged to the B/Victoria lineage in clade 1A, represented by B/Brisbane/60/2008 (the 2015/16 quadrivalent vaccine strain) (Figure 2). Viruses in this clade have N75K, N165K and S172P in their HA compared with the previous vaccine virus. Additional amino acid substitutions seen this season were I117V, N129D and V146I. A few (< 3%) UK 2015/16 B/Yamagata lineage viruses were detected, all belonging to genetic clade 3, with amino acid substitutions S150I, N165Y and G229D relative to a previous vaccine strain. More recent substitutions observed this season included N116K, K298E, E312K and also L172Q seen in the majority of B/Yamagata clade 3 viruses.

Of 99 influenza B viruses isolated from sentinel sources between December 2015 and May 2016 and analysed by HI assay, 98 (99%) were characterised as belonging to the B/Victoria/2/87 lineage and were antigenically similar to B/Brisbane/60/2008, the influenza B/Victoria-lineage component of the 2015/16 northern hemisphere quadrivalent vaccines. One virus (1%) was characterised as belonging to the B/Yamagata/16/88-lineage and was antigenically similar to B/Phuket/3073/2013, the influenza B/Yamagata-lineage component of the 2015/16 northern hemisphere trivalent and quadrivalent vaccines.

### Model fitting for vaccine effectiveness estimation

The variables incorporated in the multivariable model (month of sample collection, age group, sex, surveillance scheme and primary school programme area) were all significantly associated with swab positivity, and all except primary school programme area and sex were confounders for the vaccine effects (changed estimates by more than 5%). As with previous seasons' analyses [5-7], risk group was not included in the final model as it was not a confounder and data were missing for 119 samples (3.1%).

The crude and adjusted VE estimates against all confirmed influenza, influenza A(H1N1)pdm09 and influenza B for the 2015/16 season are given in Table 3. There were inadequate numbers to estimate VE against influenza A(H3N2). The adjusted VE was 52.4% (95% confidence interval (CI): 41.0–61.6) against all laboratory-confirmed influenza for all ages.

**Table 3:** Samples positive (cases; n = 1,155) and negative (controls; n = 2,686) for influenza A and B according to vaccination status and vaccine effectiveness estimates, United Kingdom, October 2015–May 2016

	Cases		Controls		Crude VE (95% CI)	Adjusted^a^ VE (95% CI)
	Vaccinated	Unvaccinated	Vaccinated	Unvaccinated		
Influenza A or B	165	990	727	1,959	55.1 (45.9–62.7)	52.4 (41.0–61.6)
Influenza A(H1N1)	112	658	727	1,959	54.1 (43–63.1)	54.5 (41.6–64.5)
Influenza A/6B1^b^	45	232	651	1,739	48.2 (28.8–62.8)	48.9 (26.4–64.5)
Influenza B	43	308	727	1,959	62.4 (47.7–73.0)	54.2 (33.1–68.6)
Influenza B/Victoria^b^	21	161	651	1,739	65.2 (44.6–78.1)	57.3 (28.4–74.6)

CI: confidence interval; RCGP: Royal College of General Practitioners Research and Surveillance Centre; VE: vaccine effectiveness.

^a^ Adjusted for age group, sex, month, pilot area and surveillance scheme.

^b^ Based only on data from RCGP and Scotland only.

Table 3 shows that the adjusted VE was 54.5% (95% CI: 41.6–64.5) against influenza A(H1N1)pdm09 and specifically 48.9% (95% CI: 26.4–64.5) for clade 6B1 viruses. The age-specific VE against influenza A(H1N1)pdm09 ranged from 48.5% (95% CI: 8.5–71.0) in those aged two to 17 years to 59.8% (95% CI: 35.8–74.8) in those aged 18 to 44 years (Table 4). There was no significant difference in VE against influenza A(H1N1)pdm09 by time since vaccination or period of vaccination (Table 4), overall or by age (adult/child).

**Table 4:** Adjusted vaccine effectiveness estimates for influenza by age, time since vaccination, vaccination period and risk group, United Kingdom, October 2015–May 2016 (n = 3,841)

Factor	Level	Adjusted VE^a^ by type % (95% CI)		
		A + B	A(H1N1)pdm09	B
Age (years)**^b^**	2–17	60.6 (34.4–76.3)	48.5 (8.5–71.0)	76.5 (41.9–90.5)
	18–44	55.3 (34.2–69.6)	59.8 (35.8–74.8)	45.9 (1.0–70.4)
	45–64	55.4 (34.6–69.5)	58.6 (36.9–72.8)	65.0 (15.1–85.6)
	≥ 65	29.1 (−34.1 to 61.8)	56.1 (7.2–79.3)	-20.2 (−259.1 to 59.8)
Period of vaccination**^b^**	Oct - Jan	50.0 (27.6–65.4)	54.3 (31.6–69.4)	35.9 (−70.5 to 75.9)
	Feb - April	53.0 (38.7–64.0)	53.6 (36.1–66.3)	56.9 (35.1–71.3)
Time from vaccination to onset**^b^**	< 3 months	51.4 (29.9–66.3)	56.7 (34.9–71.3)	53.1 (−12.1 to 80.3)
	> 3 months	52.7 (39.2–63.2)	53.9 (38.1–65.6)	53.4 (30.0–69.0)

CI: confidence interval; VE: vaccine effectiveness.

**^a^** Adjusted for age group, sex, month, pilot area and surveillance scheme.

**^b^** No significant evidence of interaction.

Table 3 also shows that the adjusted VE was 54.2% (95% CI: 33.1–68.6) against influenza B and specifically 57.3% (95% CI: 28.4–74.6) for viruses of the B/Victoria lineage. The age-specific VE against influenza B ranged from 76.5% (95% CI: 41.9–90.5) in those aged two to 17 years to −20.0% (95% CI: −259.1 to 59.8) in those aged 65 years and older (Table 4), although these age-specific differences in VE were not significant. There was no significant difference in influenza B VE by time since vaccination or by period of vaccination (Table 4).

The VE results by type of vaccine in children two to 17 years of age are given in Table 5. For children receiving LAIV, the overall VE against all laboratory-confirmed influenza was 57.6% (95% CI: 25.1–76) and specifically 81.4% (95% CI: 39.6–94.3) for influenza B and 41.5% (95% CI: −8.5 to 68.5) for influenza A(H1N1)pdm09. This compares to an overall VE of 77.8% (95% CI: 7.3–94.7) for children receiving IIV and a specific VE of 56.3% (95%CI: −121.6 to 91.4) against influenza B and 100% (95%CI: 13.3–100) against influenza A(H1N1)pdm09. By age group, overall LAIV VE in two to eight year-olds was 50.2% (95% CI: 1.6–74.8) and 63.9% (95% CI: −20.3 to 89.2) in nine to 17 year olds.

**Table 5:** Vaccine effectiveness estimates for influenza by type of vaccine in two to 17 year-olds, United Kingdom, October 2015–May 2016 (n = 729)

Type/subtype	Type of vaccine	Cases (unvaccinated; vaccinated)	Controls (unvaccinated; vaccinated)	Crude VE (95% CI)	Adjusted VE^a^ (95% CI)
All	Intranasal	212; 26	402; 89	44.6 (11.6–65.3)	57.6 (25.1–76)
	Injectable	212; 3	402; 16	64.4 (−23.4 to 89.8)	77.8 (7.3–94.7)
Influenza A/(H1N1)pdm09	Intranasal	112; 22	402; 89	11.3 (−47.9 to 46.8)	41.5 (−8.5 to 68.5)
	Injectable	112; 0	402; 16	100 (13.3­–100)	100 (13.3–100)^b^
Influenza B	Intranasal	95; 4	402; 89	81 (46.9–93.2)	81.4 (39.7­–94.3)
	Injectable	95; 3	402; 16	20.7 (−177.8 to 77.3)	56.3 (−121.6 to 91.4)

CI: confidence interval; VE: vaccine effectiveness.

**^a^** Adjusted for age group, sex, month, pilot area and surveillance scheme.

^b^ Cornfield's unadjusted estimate.

In 2013/14, the dominant circulating strain was influenza A(H1N1)pdm09, whereas in 2014/15, the dominant circulating strain was influenza A(H3N2), which had antigenically and genetically drifted from the vaccine strain, followed by influenza B mainly of the B/Yamagata lineage. Over the three seasons, the overall VE of LAIV was 53.1% (95% CI: 31.4–67.9) against all confirmed influenza, with a VE of 31.5% (95%CI: −50.4–68.8) for IIV (Table 6). The LAIV VE showed evidence of significant VE against laboratory-confirmed influenza B infection, borderline significance against influenza A(H3N2) and moderate, non-significant effectiveness against influenza A(H1N1)pdm09. Over the three-year period, albeit with small numbers, there was no evidence of significant effectiveness of IIV against influenza B or A(H3N2), but effectiveness of 100% (95% CI: 16.2–100) against influenza A(H1N1)pdm09.

**Table 6:** Three-season vaccine effectiveness estimates for influenza by type of vaccine in two to 17 year-olds, United Kingdom, October 2013–May 2016 (n = 1,655)

Type/subtype	Type of vaccine	Cases (unvaccinated; vaccinated)	Controls (unvaccinated; vaccinated)	Crude VE (95% CI)	Adjusted VE^a^ (95% CI)
All	Intranasal	414; 49	1,003; 189	37.2 (12.2–55)	53.1 (31.4–67.9)
	Injectable	414; 11	1,003; 29	8.1 (−85.7 to 54.5))	31.5 (−50.4 to 68.8)
Influenza A(H3N2)	Intranasal	129; 13	1,003; 189	46.5 (3.4–70.4)	46.7 (−6.9 to 73.4)
	Injectable	129; 5	1,003; 29	-34.1 (−252.4 to 49)	-22.0 (−274.8 to 60.3)
Influenza A/(H1N1)pdm09	Intranasal	159; 32	1,003; 189	-6.8 (−61 to 29.1)	35.6 (−4.4 to 60.3)
	Injectable	159; 0	1,003; 29	100 (16.2–100)	100 (16.2–100)^b^
Influenza B	Intranasal	125; 4	1,003; 189	83 (63.5–93.8)	86.9 (61.0–95.6)
	Injectable	125; 5	1,003; 29	-38.3 (−263.9 to 47.4)	24.8 (−153.3 to 77.7)

CI: confidence interval; VE: vaccine effectiveness.

**^a^** Adjusted for age group, sex, month, pilot area, surveillance scheme and year.

^b^ Cornfield's unadjusted estimate.

## Discussion

In the 2015/16 season, the UK completed the third season of the introduction of a universal paediatric LAIV programme. The 2015/16 season was characterised by late, prolonged influenza A(H1N1)pdm09 activity, with predominance of an emerging genetic HA subgroup, which was antigenically well matched to the vaccine strain, followed by circulation of influenza B viruses, predominantly of the B/Victoria lineage which was not represented in the 2015/16 trivalent inactivated influenza vaccine. The end-of-season VE was moderately good in adults for influenza A(H1N1)pdm09 and in adults younger than 65 years for influenza B, despite the B lineage mismatch for the trivalent influenza vaccine, the main vaccine used in adults. Overall VE for LAIV in children was also moderately good and specifically for influenza B, it was very good, although protection was less against influenza A(H1N1)pdm09. There was no evidence to suggest waning vaccine-derived protection or changes in circulating strains over the 2015/16 season.

We found an overall significant VE of 52.4% and specifically of 54.5% against influenza A(H1N1)pdm09, the dominant circulating strain this season. Although 2015/16 has seen the continued emergence of the new genetic subgroups 6B.1 and 6B.2, the antigenic characterisation indicates a good match to the 2015/16 influenza vaccine strain and no measurable differences between these two emerging groups, which reinforces the VE findings in this paper. These levels of effectiveness are consistent with those reported mid-season in 2015/16 [5], but also in earlier A(H1N1)pdm09 seasons, in particular in 2010/11 [14]. The 2015/16 A(H1N1)pdm09 VE results were also similar to the mid-season estimates reported from North America and elsewhere in Europe this season [15,16]. The continuing apparent antigenic and epidemiological match to the vaccine strain remains encouraging and supports the World Health Organization’s recommendation that the vaccine for the 2016/17 northern hemisphere winter should include an A/California/7/2009-like vaccine strain [17].

In younger adults under 65 years of age, influenza B VE was over 50%. Almost all vaccinated adults in the UK can be expected to have received the 2015/16 trivalent inactivated (rather than the quadrivalent) influenza vaccine, which contained the B/Yamagata vaccine strain in 2015/16. Our results indicate that despite this lineage mismatch, the 2015/16 IIV in younger adults continued to provide important levels of protection against influenza B, findings which are consistent with earlier published literature [18]. On the other hand, we failed to find evidence of significant VE against influenza B in the elderly, although underpowered with only 19 positive detections and a low positivity of 4.6% in this age group. This is in contrast to the 2014/15 season, when influenza vaccines elsewhere in Europe provided effectiveness of 50.4% (95% CI: 14.6–71.2) against influenza B in those older than 65 years [19]; in that season, the dominant circulating strain was B/Yamagata and belonged to a clade that was antigenically similar to the vaccine virus that season. Evidence of cross-protection, as we seem to have seen in the younger adults this season, might have important implications for the potential incremental cost-effectiveness and recommendations for preferential use of quadrivalent vaccines in adults and highlights the importance of gathering further data in this area to better inform such decisions.

Among children two to 17 years of age, we observed an overall significant VE of 57.6% for the quadrivalent LAIV vaccine this season, specifically 81.4% for influenza B and 41.5% for influenza A(H1N1)pdm09, with a similar picture when examining the previous three seasons. Over the three seasons, the overall effectiveness of LAIV was higher compared with inactivated vaccine in that age group, specifically for influenza A(H3N2) and B, but lower in 2015/16 and specifically for influenza A(H1N1)pdm09. These findings are in contrast to those recently reported by the US CDC who found an overall VE of only 3% for LAIV in two to 17 year-old children with very low VE against influenza A(H1N1)pdm09, while the inactivated vaccine showed significant effectiveness [3]. The US first noted lower VE of LAIV against influenza A(H1N1)pdm09 in 2013/14, which on further investigation was considered related to reduced thermostability of the A/California/7/2009 vaccine strain [20]. This led to the replacement of the A(H1N1)pdm09 LAIV vaccine strain with the more recently emerged A/Bolivia/559/2013 vaccine strain for the 2015/16 season. Based on the 2015/16 VE findings from the CDC, the US Advisory Committee on Immunisation recommended a temporary suspension of use of LAIV for children in the US for the forthcoming 2016/17 season [3]. In addition to the UK findings presented here, Finland, in its first season of use of LAIV in pre-school age children, found overall levels of protection of 51%, similar to the UK [21]. 

The reasons why the observed levels of overall protection were higher in Europe than in the US, with apparent reduced protection against influenza A(H1N1)pdm09 compared to IIV, remain under investigation. Several hypotheses have been suggested. Firstly, are the observed differences real or the consequence of a methodological difference? If real, viral interference between the A(H1N1)pdm09 vaccine strain and the other influenza vaccine viruses in the quadrivalent LAIV vaccine might provide an explanation; such interference has been discussed previously [22] and might be reinforced by prior vaccination with LAIV and/or IIV in young children (which is at present much more likely in North America than Europe) or by repeat vaccination in-season, with the US offering two doses of influenza vaccine to children compared with one dose for healthy children in Europe. A further explanation is possible antigenic drift between the A/Bolivia/559/2013 vaccine strain in the 2015/16 LAIV vaccine and circulating A(H1N1)pdm09 strains in winter 2015/16, although antigenically, the virus is considered to be well matched. Finally, programmatic or logistical differences, e.g. related to cold chain or vaccine handling might play a role. 

Further work is required to investigate these hypotheses, although UK programme evaluation results from 2013/14 and 2014/15 already suggest that the UK LAIV paediatric programme reduced influenza circulation when comparing pilot areas where children of primary school age were offered vaccine to those areas where they were not [23,24]. The UK VE results presented in this paper have been reviewed by the JCVI who strongly recommended not to change the current influenza immunisation strategy planned for 2016/17, but further work is required to better understand these recent observations in the light of the US findings and to potentially optimise vaccine composition.

Although waning protection post vaccination has recently been noted [25] and although 2015/16 was a particularly late influenza season with significant activity until late into the spring, there was no evidence to suggest either waning protection by time since vaccination or changes in effectiveness by vaccination period due to the emergence of new clades or lineages over the course of the season in the UK. Our findings are congruent with recent work which suggests that intra-seasonal waning is of lesser importance with influenza A(H1N1)pdm09 and influenza B compared with influenza A(H3N2) [25].

The paper has a number of strengths. It uses a well-established methodology, the TNCC, the results of which approximate well to more traditional case–control approaches [26]. Data completeness was very high and the integration of genetic characterisation data has allowed the estimation of clade- and lineage-specific VE. Caution is needed in the interpretation of the results in children two to 17 years of age owing to the small sample size, particularly in relation to IIV where only a small proportion of the paediatric control population with available information (16/507, 3%) were reported to be vaccinated, while for LAIV, 18% of controls were reported vaccinated.

## Conclusion

In summary, notwithstanding the limitation of the small sample size, our findings together with those from Finland confirm encouraging overall levels of protection for LAIV. This protection is particularly effective against influenza B, though less against influenza A(H1N1)pdm09, a finding which in the light of observations in the US requires further investigation.
